# The effect of instructed refreshing on working memory: Is the memory boost a function of refreshing frequency or refreshing duration?

**DOI:** 10.3758/s13421-024-01666-w

**Published:** 2024-12-02

**Authors:** Evie Vergauwe, Alessandra S. Souza, Naomi Langerock, Klaus Oberauer

**Affiliations:** 1 Faculté de Psychologie et des Sciences de l’Education, Université de Genève, 40 Bd du Pont d’Arve, 1211 Geneva 4, Switzerland; 2 Department of Psychology, University of Zürich, Zurich, Switzerland; 3 Center for Psychology, Faculty of Psychology and Education Sciences, University of Porto, Porto, Portugal

**Keywords:** Working memory, Focus of attention, Refreshing

## Abstract

Refreshing is assumed to reactivate the contents of working memory in an attention-based way, resulting in a boost of the attended representations and hence improving their subsequent memory. Here, we examined whether the refreshing-induced memory boost is a constant or a gradual, time-dependent phenomenon. If the beneficial effect of refreshing on memory performance is due to the information being selected for refreshing (i.e., selection hypothesis), a constant memory boost is expected to occur each time an item is selected for refreshing, with better memory performance for items that are selected more often. If, however, the beneficial effect of refreshing on memory performance is due to spending time in the focus of attention during refreshing (i.e., duration hypothesis), a gradual memory boost is expected, with the size of the memory boost being a direct function of how long the item has been the object of focused attention. To distinguish between these hypotheses, we instructed and guided the use of refreshing during retention through the presentation of cues, and varied the number of refreshing steps and their duration independently. The number of refreshing steps, but not their duration, had an effect on recall, in agreement with the selection hypothesis. However, some of the results were less robust than anticipated, indicating that the effect of instructed refreshing is limited to certain task parameters.

**Supplementary Information:**

The online version contains supplementary material available at 10.3758/s13421-024-01666-w.

## Introduction

The ability to keep information in mind over the short term is crucial for cognition. The cognitive system devoted to short-term maintenance of relevant information is called working memory, and an ongoing debate centers around the role and the nature of processes involved in keeping information active in working memory. Refreshing is one of the key processes that has been proposed to support short-term maintenance, using attention to reactivate the contents of working memory (see Camos et al., 2018, for a review). Here, we ask how the act of refreshing results in better working memory for the refreshed information.

### Refreshing results in a memory boost

Refreshing has been proposed as a domain-general maintenance process in working memory, relying on central attention to reactivate the content of working memory (e.g., Barrouillet et al., 2007; Johnson, 1992; Souza et al., 2018; Vergauwe et al., 2014). During refreshing, working memory representations are assumed to be reactivated by the focus of attention being directed at them, one by one, in their order of presentation (e.g., Barrouillet & Camos, 2012; Cowan, 1995; Higgins & Johnson, 2009; Vergauwe & Cowan, 2014; but see Portrat & Lemaire, 2015, for an alternative view). Being brought into the focus of attention is assumed to increase the accessibility of the information in question, resulting in a memory boost for the refreshed information. In line with the notion that refreshing results in increased accessibility of the refreshed information, recent studies have shown that, right after refreshing a particular memory item, response times are particularly fast for memory probes that match the just-refreshed memory item, for both verbal and visuo-spatial memory materials (Vergauwe & Langerock, 2017, 2024).

At least two findings in the literature are consistent with the notion that refreshing results in a memory boost. First, a series of studies has shown that reducing the proportion of time during which refreshing of a memory list can take place results in poorer recall performance for that list. Typically, these studies use complex span tasks in which a list of memory items is presented and each memory item is followed by a brief delay during which a secondary processing task is to be performed. The observation is that, as the secondary task requires attention for a larger proportion of time (e.g., because the task is more complex, or because there is more information to be processed), recall performance for the memory list decreases, for both verbal and visuo-spatial memory materials (e.g., Barrouillet et al., 2004, 2007, 2011; Vergauwe et al., 2009, 2010, 2012; but see Langerock et al., 2025; Ricker & Vergauwe, 2020, 2022; Schneider et al., 2023). This effect is referred to as the cognitive load effect, and is often interpreted as evidence for the spontaneous occurrence of refreshing. It is assumed that when the secondary task requires attention for a larger proportion of the delay time, there is less time for refreshing and, as a result, memory is poorer.

A more direct test of the mnemonic consequences of refreshing was provided by Souza and colleagues (2015). These authors aimed to bring refreshing under experimental control by instructing participants to refresh certain items during retention. Participants were presented with an array of colored disks, and during the retention interval that separated the array presentation from recall, refreshing cues were presented. These cues consisted of arrows pointing to some of the locations where a colored disk had been presented, and participants were instructed to “think of” the color that had been presented in each of the cued locations. On each trial, there were some items that had been refreshed once, some items that were refreshed twice, and some items that had not been refreshed at all. This allowed the authors to demonstrate that recall performance was a function of refreshing frequency, with smaller recall error for items that had been refreshed more often. The observation of a monotonic decrease in recall error as a function of increasing refreshing frequency has been replicated a few times, for both verbal and visuo-spatial memory materials (Atkinson et al., 2022; Loaiza & Souza, 2022; Souza & Oberauer, 2017; Souza et al., 2018), and demonstrates that refreshing results in a cumulative memory boost for items refreshed more often. It is currently unclear, however, how the act of refreshing results in better working memory for the refreshed information. In particular, we do not know whether the cumulative memory boost induced by refreshing occurs because of multiple constant boosts applied to the refreshed items or whether the cumulative memory boost is the result of a gradual strengthening of these items as they remained longer in the focus of attention.

### Is the refreshing-induced memory boost a constant or a gradual phenomenon?

A constant versus gradual memory boost reflects two different ways in which refreshing may operate on the contents of working memory. A constant boost is expected if the beneficial effect of refreshing stems from a fixed memory boost being applied to an item every time it is selected for attentional focusing (i.e., the *selection hypothesis*). This boost could occur through various mechanisms. For example, entering the focus of attention might simply increase the activation level of the cued item, or it could strengthen the binding between the cued item and its context (e.g., its location; see Loaiza & McCabe, 2012; Rerko et al., 2014). Regardless of the specific mechanism, according to the selection hypothesis, a constant memory boost occurs each time an item is selected for refreshing. Thus, as an item is selected more often for refreshing, more memory boosts accumulate, resulting in better memory for these items. This implies that the main determinant of memory performance is the number of times an item has been refreshed, as originally proposed by Souza and collaborators (Souza et al., 2015, 2018).

In contrast, a gradual boost is expected if the beneficial effect of refreshing is time-dependent, such that the size of the memory boost is a direct function of how long the item has been the object of focused attention (i.e., the *duration hypothesis*). According to the duration hypothesis, spending time in the focus of attention results in the continuous activation and gradual strengthening of the representation, with the strengthening being proportional to the duration of the focusing event. This implies that the main determinant of memory performance is the total amount of time during which an item has been refreshed. This hypothesis would account for the findings of Souza and colleagues by proposing that items that were cued more often to be refreshed, spent more time in the focus of attention overall, resulting in a larger memory boost.

The effect of cognitive load on memory performance is compatible with both hypotheses, because the cognitive load effect only shows that more available time for refreshing results in better memory. It is not known how people allocate that time to attend to each memory item (if they do so at all). In line with the frequency hypothesis, longer periods of free time could be used to refresh each item more often for a fixed amount of time. In line with the duration hypothesis, longer periods of free time (i.e., time during which attention is not used for processing) could be used to refresh each item for longer. Both scenarios would lead to improved memory performance in conditions with a lower cognitive load.

Studies using cued refreshing (e.g., Souza & Oberauer, 2017; Souza et al., 2015, 2018) have more experimental control over the studied process. However, the selection versus duration hypotheses cannot be disentangled in the existing studies with this paradigm either, because the number of refreshing events and the total duration of refreshing are confounded. This is because items were cued to be refreshed 0, 1, or 2 times, with each refreshing event having a duration of 500 ms. An item that had been refreshed once had a total refreshing duration of 500 ms, whereas an item that had been refreshed twice had a total refreshing duration of 1,000 ms, making it impossible to determine whether the observed memory boost resulted from items being selected more often or from items being refreshed for a longer total amount of time. To distinguish between the selection and duration hypotheses, one needs to vary the number of refreshing steps and their duration independently. That is what we did in the current study.

## The current study

We examined the effects of refreshing frequency and refreshing duration on memory performance in three experiments. In all three experiments, participants needed to remember the hue of six colored disks, and some items were cued to be refreshed during retention. In Experiment 1, items were cued to be refreshed 0, 1, or 2 times, and each refreshing event had a duration of either 500 ms (like in Souza et al., 2015, 2018) or 1,000 ms. We expected to replicate the findings of Souza and colleagues, with a smaller recall error for items that were refreshed more frequently. Importantly, the duration hypothesis predicted a beneficial effect of refreshing duration as well, with longer refreshing steps yielding a smaller recall error. To anticipate, the results of Experiment 1 were in line with the predictions of the selection hypothesis but not with the predictions of the duration hypothesis; increasing the number of refreshing steps had an impact on memory performance, whereas increasing their duration did not.

However, one possibility is that the time an item spends in the focus of attention does impact the resulting memory boost (as predicted by the duration hypothesis), but not beyond a duration of 500 ms (see literature on the retro-cue effect, described below). To address this possibility, in Experiment 2, items were again cued to be refreshed 0, 1, or 2 times, but each refreshing event had now a duration of either 250 or 500 ms. In Experiment 3, items were cued for 0, 1, 2, or 3 times, each refreshing event having a duration of 333, 500, or 1,000 ms. Overall, the findings of these two additional experiments indicate that recall performance is not dependent on the duration of the refreshing steps.

## Experiment 1

### Method

#### Participants and design

For all studies reported here, participants had normal or corrected-to-normal vision. They provided signed informed consent before participating. Debriefing was available to all participants. The experimental protocol used in all experiments was approved by the ethics committee of the Faculty of Psychology and Educational Sciences at the University of Geneva. Thirty undergraduate students from the University of Geneva participated for partial course credit. Refreshing frequency (three levels: 0, 1, or 2 refreshing steps) and Duration of refreshing steps (two levels: 500 vs. 1,000 ms) were manipulated as two within-subjects variables. The sample size was based on previous studies using the cued refreshing paradigm (e.g., Souza et al., 2015, 2018).

#### Materials and procedure

**Fig. 1:**
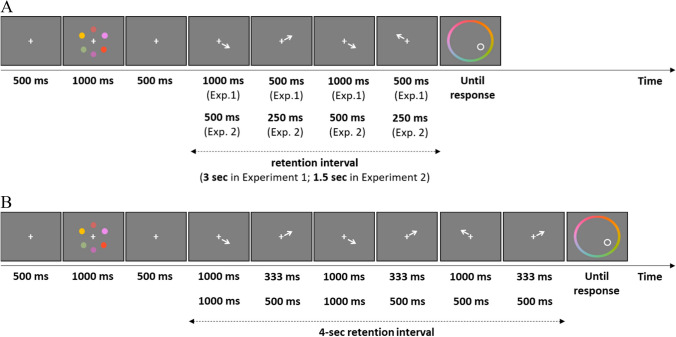
Schematic illustration of a trial in Experiments 1 and 2 (**panel A**), and in Experiment 3 (**panel B**)

Participants performed a continuous delayed estimation task in which six colors were to be remembered (see Fig. 1A). The experiment was programmed using MATLAB and the Psychophysics Toolbox extension (Brainard, 1997; Pelli, 1997). Participants were tested in individual booths, and were seated such that they could comfortably view the screen (viewing distance unconstrained). All stimuli were presented on a standard computer screen against a gray background. Our procedure was similar to the one developed by Souza et al. (2015). In particular, participants were asked to memorize six colors, and to reproduce a randomly probed color after a retention interval by clicking on a color wheel. The colors used as memoranda were sampled from a continuous CIE L*a*b color model (L = 70, a = 20, b = 38, and radius = 60) with 360 values evenly distributed along a color wheel (cf. Zhang & Luck, 2008). On a given trial, colors were selected randomly with the constraint that all six colors were at a minimum distance of 20° of each other on the color wheel.

Figure 1A illustrates the sequence of events in each trial. A trial started with a white fixation cross displayed in the center of the screen for 500 ms, followed by the presentation of six colored disks for 1,000 ms. Next, after a 500-ms pre-cue time, a 3-s retention interval started during which different locations were cued to be refreshed. Participants were instructed to think of the color that appeared in the cued location for as long as the cue was on-screen. At the end of the retention interval, one of the colored disks had to be reproduced: a test display was shown containing a color wheel (randomly rotated from trial to trial), a white circle frame (indicating the location of the target item), and a question (“Color?” in French). Participants were asked to indicate the color of the target with a mouse click on the color wheel. The next trial started 1 s later. Instructions emphasized accuracy but not speed. To minimize the use of verbal encoding and articulatory rehearsal, participants were asked to repeat the sequence "babibou" aloud throughout the experiment.

Different colors were cued for refreshing during the retention interval. Specifically, four arrows served as cues and were presented sequentially in the center of the screen. Each of these arrow cues represents one refreshing step, and remained on-screen for either 500 or 1,000 ms. As in Souza et al. (2015), participants were instructed to think of the color the arrow pointed to. Participants were told that some arrows would appear for a short period of time while other arrows would remain on-screen a bit longer. Participants were told that the cues did not reliably indicate the item to be tested, but that thinking of the cued color for as long as the arrow remains on-screen is part of their main task. There were ten possible cue sequences. In terms of which items were to be refreshed, we used the same five possible cue schedules as in Souza et al. (2015): the four arrows could point to four different items (A-B-C-D), they could point to two different items once and to a third item twice (A-B-A-C; A-B-C-B; A-B-C-A), or to two different items twice (A-B-A-B). Thus, across two successive cues, different items were always cued. Using these five cue schedules, we manipulated the refreshing frequency of the target color: 0, 1, or 2 refreshing steps. That is, given the described constraints, there were, on average, three items that were not cued (0-Refreshing items), two items that were cued once (1-Refreshing items), and one item that was cued twice (2-Refreshing items), in each memory array.

During each 3-s retention interval, two items were cued over 500 ms and two items were cued over 1,000 ms. The total duration of the cuing interval was thus kept constant at 3,000 ms. To determine how long each refreshing step takes, two versions of each of the previously described possible cue schedules were created. For each of the five possible cue schedules, these two versions were used equally often (randomly intermixed). When the cue sequence A-B-C-A was used, the items that were cued for 1,000 ms were either the two inner items (B and C) or the two outer items (A), the remaining items being cued for 500 ms. For the four other cue schedules, the items that were cued for 1,000 ms were either the first and third items, or the second and fourth items, the remaining items being cued for 500 ms. This procedure guaranteed that when an item was selected to be refreshed twice, it was refreshed for the same duration in each of the steps. The resulting ten cue sequences (two versions of the five different cue schedules) are described in more detail in Supplementary Table S1 (see Online Supplementary Material (OSM)). Note that the number of attention switches between different items was kept constant at three in each of these ten sequences.

Each participant performed 300 trials. In an equal proportion of trials (randomly intermixed), the target of recall was selected to be a 0-refreshing item (i.e., 100 trials per participant), a 1-refeshing item (i.e., 100 trials per participant, with approximately half being 1*500 ms-refreshing items and the other half being 1*1,000 ms-refreshing items), or a 2-refreshing item (i.e., 100 trials per participant, with approximately half being 2*500 ms-refreshing items and the other half being 2*1,000 ms-refreshing item items). In the beginning of the session, participants completed six practice trials that were discarded from subsequent analyses.

#### Data analysis

For all experiments reported here, a raw recall error score was calculated for each trial by computing the absolute distance between the position of the target color and the position of the response color (i.e., recall error). This value can range from 0 (perfect recall) to 180 (recall at the opposite location to the correct one). Recall error served as our dependent variable. For each experiment, two main analyses are reported, both using Bayesian ANOVAs. In the first main analysis, recall error was analyzed as a function of the number of refreshing steps, to assess if we were able to replicate the refreshing frequency effect reported previously in the literature. In the second main analysis, we assessed the evidence for the effects of refreshing frequency versus refreshing duration. The data were analyzed in R (R core team, 2022; version 4.1.3), using the BayesFactor package (Morey et al., 2022; version 0.9.12–4.4). We used the default prior settings available in the package. We used Bayesian statistics to assess the strength of evidence in the data for the presence or absence of an effect of our manipulations, which is represented by the Bayes factor (BF). BFs can be reported in favor of the alternative hypothesis that there is an effect (BF_10_) or in favor of the null hypothesis that there is no effect (BF_01_; which is simply 1/BF_10_). BFs between 1 and 3 were considered ambiguous. BFs > 3, but below 10, were considered to show moderate evidence for an effect. BFs > 10 were considered to indicate strong evidence.

Additional analyses used Bayesian t-tests to test whether memory improved with an increased number of refreshing steps when the total duration of refreshing was held constant. In Experiment 3, we also tested whether longer refreshing durations improved memory when the number of refreshing steps was held constant. In the Online Supplementary Material (OSM), we report further analyses addressing a potential confound between the number of refreshing steps and the delay between the last refreshing cue and the test. All effect sizes were calculated using JASP (JASP team, 2020).

### Results

**Fig. 2:**
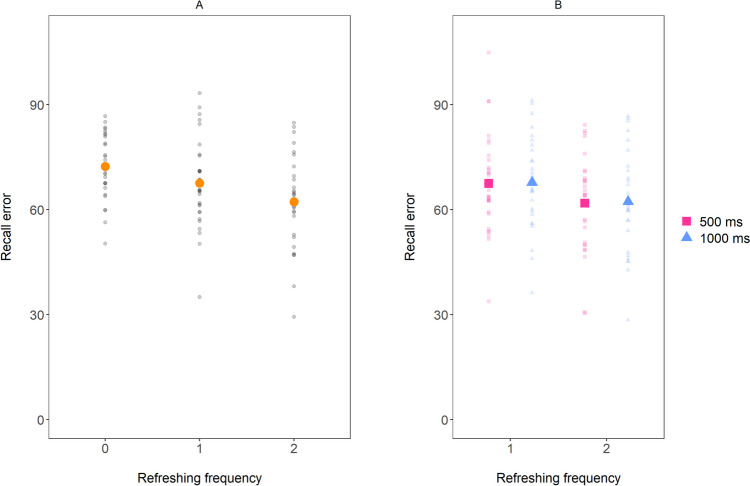
Mean recall error in Experiment 1, as a function of Refreshing frequency (including 0 refreshing) in **panel A** and as a function of Refreshing frequency (excluding 0 refreshing) and Duration of refreshing steps in **panel B**. The small dots represent the data of individual participants. The larger dots represent the sample mean

First, recall error was aggregated per participant and per level of Refreshing frequency (0, 1, or 2 refreshing steps). Mean performance is shown in Fig. 2A. We submitted these data to a Bayesian ANOVA with Refreshing frequency (0, 1, or 2 refreshing steps) as within-subject predictor, which revealed strong evidence for a main effect of the number of refreshing steps on recall (BF_10_ = 926.63; η^2^ = 0.31). We thus replicate the findings by Souza et al., (2015; see also Loaiza & Souza, 2022; Schneider et al., unpublished), with better recall for items that have been refreshed more often.

Next, we examined whether the duration of each refreshing step has an effect on recall error. The relevant data are shown in Fig. 2B. Recall error does not seem to be affected by the duration of each refreshing step. A Bayesian ANOVA on mean recall error with Number of refreshing steps (1 or 2) and Duration of refreshing steps (500 vs. 1,000 ms) showed that the Number of refreshing steps-only model was the best model (BF_10_ for the main effect of Number of refreshing steps = 162.15; η^2^ = 0.15). There was modest evidence *against* including the variable Duration of refreshing steps (BF_01_ = 5.01; η^2^ < 0.01), and the Number of refreshing steps-only model was preferred over the full model (including both main effects as well as their interaction) by a factor of 19.16 (η^2^ < 0.01 associated with the interaction effect). As such, it appears that the effect of refreshing on recall error is entirely due to the number of refreshing steps, as lengthening the duration of each refreshing step from 500 to 1,000 ms did not have an effect on recall error.

Considering recall error for refreshed items only when they were last cued in the first or the second half of the cue sequences gave similar results (see Supplementary Analyses 1 and 2, OSM).

Finally, a one-sided, paired t-test was run to test the frequency hypothesis more directly (see Supplementary Analysis 3, OSM, for a similar approach regarding the duration hypothesis). Specifically, a one-sided t-test tested our directional hypothesis: whether recall error was smaller for items that had been refreshed twice for 500 ms than for items that had been refreshed once for 1,000 ms. This analysis tests the predicted positive effect of refreshing frequency while keeping the total amount of refreshing time constant. There was indeed modest evidence for this difference (BF_10_ = 6.63; d = 0.48). Similar results were obtained when only considering recall error for items that were last cued by the third or fourth cue, although the evidence was less strong (BF_10_ = 2.40; d = 0.38). Thus, even though the total duration of refreshing was the same for these two types of items (i.e., 1,000 ms), recall performance was found to be better when an item was refreshed more often.

### Discussion

Experiment 1 revealed a coherent pattern of results that provides evidence for the selection hypothesis but not for the duration hypothesis. As expected, recall performance was found to vary as a function of the number of times an item had been refreshed. Directly assessing the contribution of the number of refreshing steps versus the duration of each refreshing step showed that the memory boost induced by instructed refreshing was explained solely by the number of refreshing steps; extending the duration of each refreshing step from 500 to 1,000 ms did not affect recall error (see also Loaiza & Souza, 2022). The fact that we found evidence for the effect of the number of refreshing steps, regardless of whether the 0-refreshing condition was included in the analysis, indicates that the overall effect of the number of refreshing steps is not primarily driven by the difference in recall performance between items that had been refreshed at least once versus items that had not been refreshed (see also Supplementary Analysis 3 (OSM)). Moreover, recall performance was better for items that were refreshed twice for 500 ms, relative to items that were refreshed once for 1,000 ms, which shows a beneficial effect of refreshing frequency while the total duration of refreshing is controlled for.

One could, however, argue that the evidence against the duration hypothesis is related to the specific durations of the refreshing steps used in Experiment 1. Indeed, we extended the duration of each refreshing step from 500 to 1,000 ms, assuming that more time in the focus of attention would result in a larger boost. This should have been the case, if the duration hypothesis explains the cumulative refreshing-induced memory boost previously observed by Souza and colleagues (2015, 2018). In these studies, items refreshed twice were in the focus of attention for a total duration of 1,000 ms, and they were recalled better than items that were refreshed once and that were hence in the focus of attention for only 500 ms. The current findings rule out a duration explanation of the findings of Souza and colleagues. Instead, the current findings support a selection explanation of their results, with better memory performance for items that were more frequently selected into the focus of attention.

It is possible, however, that the memory boost associated with refreshing is gradual and dependent on how long an item remains in the focus of attention, but not beyond 500 ms. Only after that limit, the refreshing benefit would be explained by the frequency of selection in the focus of attention. Some findings in the literature appear to be consistent with the idea that 500 ms may provide an upper limit for a gradual strengthening in the focus of attention. Studies using a single cue to direct attention to one item in working memory (i.e., retro-cue studies) have observed that participants show a focusing benefit when they have at least 250–300 ms to focus attention on the cued item (Pertzov et al., 2013; Souza et al., 2014, 2016; Tanoue & Berryhill, 2012). Additionally, using a procedure to measure the spontaneous speed of refreshing in working memory, Oberauer and Souza (2020) observed that participants needed about 200 ms to select items for refreshing irrespective of the nature of the memoranda (words, colors, or pictures). Together, these studies indicate that 500 ms is ample time to select an item in the focus of attention and to strengthen it to its maximal potential in each refreshing step. Accordingly, the durations selected in Experiment 1 may have been too long to allow us to observe gradual strengthening as a function of refreshing. To test the possibility that the duration of refreshing has an impact on memory performance but only on short time scales, Experiment 2 compared refreshing durations of 250 and 500 ms, again including a manipulation of the number of times an item is refreshed (0, 1, or 2 times).

## Experiment 2

### Method

#### Participants and design

Thirty undergraduate students from the University of Geneva participated for partial course credit. None had participated in Experiment 1. Refreshing frequency (3 levels: 0, 1, or 2 refreshing steps) and Duration of refreshing steps (2 levels: 250 vs. 500 ms) were manipulated as two within-subjects variables. Like in Experiment 1, the sample size was based on previous studies using the cued refreshing paradigm (e.g., Souza et al., 2015, 2018).

#### Materials and procedure

Participants performed the same continuous delayed estimation task as in Experiment 1, except for a few changes that are detailed below (see Fig. 1A). Like in Experiment 1, four arrows served as cues and were presented sequentially in the center of the screen during the retention interval. In Experiment 2, each of these arrow cues remained on screen for either 250 or 500 ms (as opposed to 500 vs. 1,000 ms, used in Experiment 1). Participants received the same instructions as in Experiment 1, and we used the same five possible cue schedules as in Experiment 1 (A-B-C-D, A-B-A-C, A-B-C-B, A-B-C-A, and A-B-A-B).

The retention interval had a fixed duration of 1,500 ms (as opposed to 3,000 ms in Experiment 1), and two items were cued during 250 ms and two items were cued during 500 ms in this interval. The two durations were assigned to the refreshing steps in the same way as in Experiment 1.

### Results

**Fig. 3:**
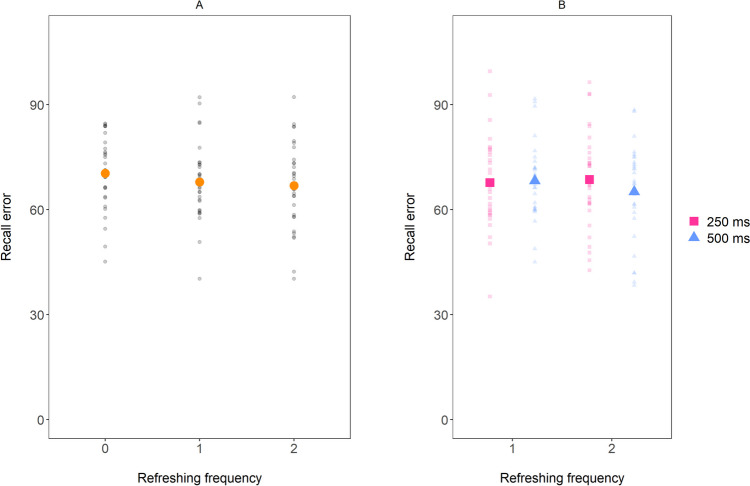
Mean recall error in Experiment 2, as a function of Refreshing frequency (including 0 refreshing) in **panel A** and as a function of Refreshing frequency (excluding 0 refreshing) and Duration of refreshing steps in **panel B**. The small dots represent the data of individual participants. The larger dots represent the sample mean

As in Experiment 1, we again started by running a Bayesian ANOVA on mean recall error with Refreshing frequency (0, 1, or 2 refreshing steps) as within-subject predictor. Although recall error appeared to be affected slightly by how often the item had been refreshed, as shown in Fig. 3A, the best model was the null model and there was some weak evidence against the main effect of Refreshing frequency (BF_01_ = 2.22; η^2^ = 0.06).

In the next step, we examined whether the duration of each refreshing step had an effect on recall error. As can be seen in Fig. 3B, recall error does not seem to be affected by the duration of each refreshing step. In fact, a Bayesian ANOVA on mean recall error with Number of refreshing steps (1 or 2) and Duration of refreshing steps (250 vs. 500 ms) revealed that the best model of the data was the null model (BF_01_ of 3.86 *against* the main effect of Number of refreshing steps, BF_01_ of 3.26 *against* the main effect of Duration of refreshing steps, and BF_01_ of 20.64 against the full model including both main effects as well as their interaction; η^2^ < 0.01 associated with the main effect of Number of refreshing steps, η^2^ = 0.01 associated with the main effect of Duration of refreshing steps, and η^2^ = 0.02 associated with the interaction effect).

Considering recall error for refreshed items only when they were last cued in the first or the second half of the cue sequences gave similar results (see Supplementary Analyses 1 and 2 (OSM)).

Finally, a one-sided, paired t-test was run, as in Experiment 1, to test the frequency hypothesis more directly (see Supplementary Analysis 3 (OSM), for a similar approach regarding the duration hypothesis). In contrast with the frequency hypothesis, recall error was not smaller for items that had been refreshing twice for 250 ms than for items that had been refreshed once for 500 ms (BF_01_ = 5.76 for the Null; d = 0.03). Similar results were obtained when only considering recall error for items that were last cued by the third or fourth cue (BF_01_ = 8.20 for the Null; d = 0.13).

### Discussion

The results of Experiment 2, in which we contrasted refreshing step durations of 250 versus 500 ms, were quite different from what we observed in Experiment 1, in which we contrasted refreshing steps of 500 versus 1,000 ms. Overall, there was no convincing evidence in the data for either the selection hypothesis or the duration hypothesis. Instead, our refreshing manipulations appear to not have had any impact on recall performance. As such, Experiment 2 did not allow us to conclude anything on the constant versus gradual nature of the memory boost induced by refreshing. One possible explanation is that including very short refreshing steps of 250 ms discouraged participants to follow and use our refreshing cues. In a further attempt to test the hypothesis by which the duration of each refreshing step has an effect on the resulting memory boost, but not beyond a time window of 500 ms, we compared refreshing step durations of 333, 500, and 1,000 ms in Experiment 3. If the duration of each refreshing step affects performance, but not beyond 500 ms, then memory performance should improve when the duration of each refreshing step is increased from 333 to 500, but not when it is increased from 500 to 1,000 ms. Together with the inclusion of refreshing steps of 333 ms, we also included items that were refreshed three times. Thus, in Experiment 3, items were cued to be refreshed 0, 1, 2, or 3 times during retention, and each refreshing step had a duration of 333, 500, or 1,000 ms.

## Experiment 3

### Method

#### Participants and design

Seventy undergraduate students (35 from the University of Geneva, and 35 from the University of Zurich) participated for partial course credit at the University of Geneva and for partial course credit or in exchange for 15 Swiss francs at the University of Zurich. The number of participants was increased, relative to Experiments 1 and 2, to compensate for the increase in the number of experimental conditions (resulting in less data per experimental cell). Refreshing frequency (4 levels: 0, 1, 2, or 3 refreshing steps) and Duration of refreshing steps (3 levels: 333, 500, or 1,000 ms) were manipulated as two within-subjects variables.

#### Materials and procedure

Participants performed the same continuous delayed estimation task as in Experiments 1 and 2, except for the following modifications (see Fig. 1B). The retention interval had a duration of 4,000 ms, and six arrow cues were presented sequentially in the center of the screen during this retention interval. The arrows remained on-screen for 333, 500, or 1,000 ms. Participants received the same instructions as in Experiments 1 and 2. Thirty-six possible cue sequences were used. These were created with the restriction that three different memory items were cued in each sequence, that two successive cues never pointed to the same memory item, and that the number of object switches was thus held constant at five across these sequences. Furthermore, these sequences were chosen such that the target items covered all possible combinations of refreshing-step durations with the number of refreshing steps of the target item. A detailed overview of the cue sequences can be found in Supplementary Table S2 (OSM).

Each participant performed 300 trials. In an equal proportion of trials (randomly intermixed), the target of recall was selected to be a 0-refreshing item (i.e., 75 trials per participant), a 1-refeshing item (i.e., 75 trials per participant, with approximately one-third for each of the refreshing durations), a 2-refreshing item (i.e., 75 trials per participant, with approximately one-third for each of the refreshing durations), or a 3-refreshing item (i.e., 75 trials per participant, with approximately one-third for each of the refreshing durations). In the beginning of the session, participants completed 12 practice trials that were not included in analyses.

### Results

**Fig. 4:**
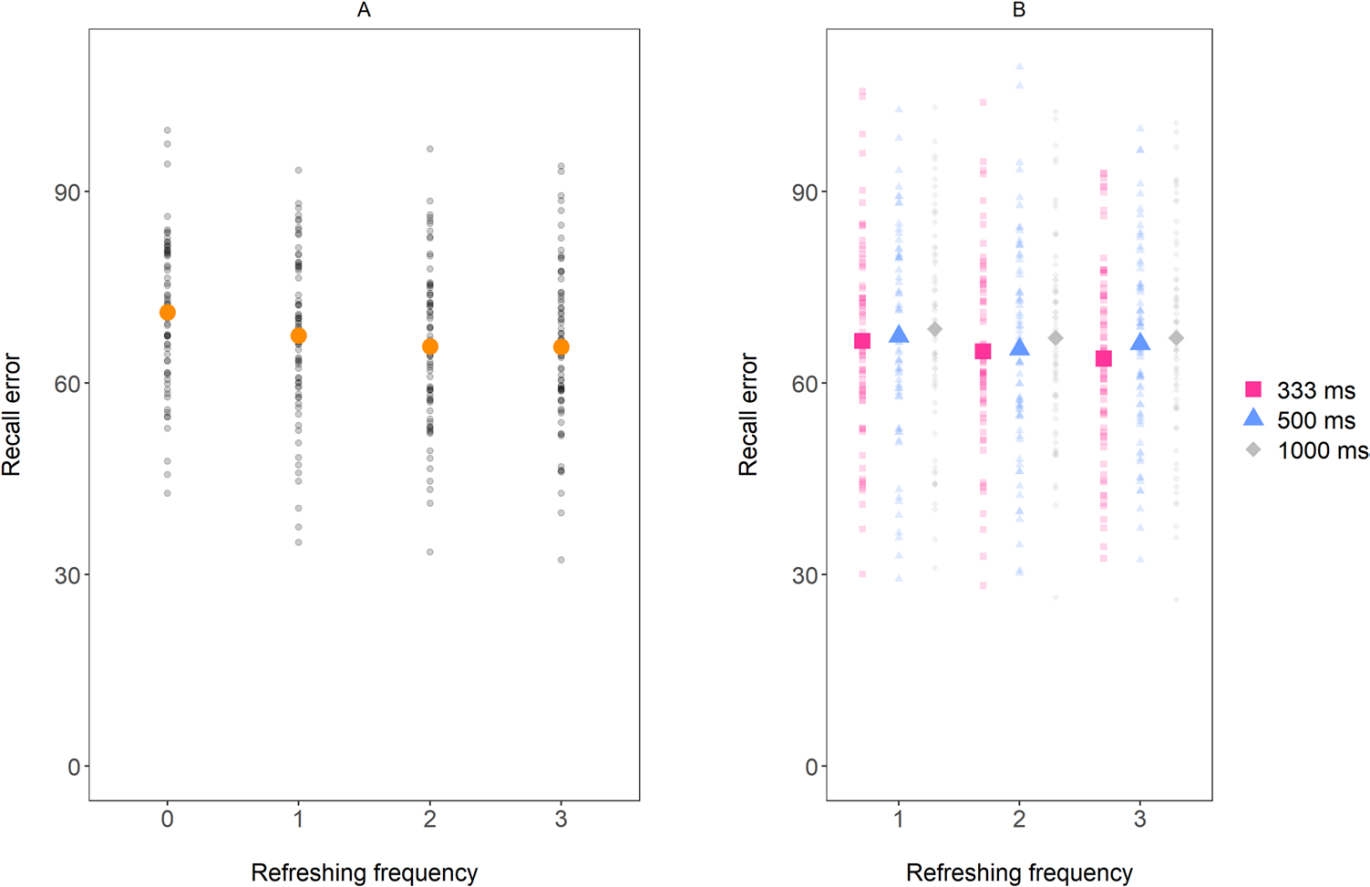
Mean recall error in Experiment 3, as a function of Refreshing frequency (including 0 refreshing) in **panel A** and as a function of Refreshing frequency (excluding 0 refreshing) and Duration of refreshing steps in **panel B**. The small dots represent the data of individual participants. The larger dots represent the sample mean

Figure 4 A shows recall error as a function of the number of refreshing steps. A Bayesian ANOVA with Refreshing frequency (0, 1, 2, or 3 refreshing steps) as within-subject predictor revealed strong evidence for a main effect of the number of refreshing steps on recall error (BF_10_ = 2769.89; η^2^ = 0.12), replicating the findings of Experiment 1.

In the next step, we examined whether the duration of each refreshing step has an effect on recall error. As can be seen in Fig. 4B, recall error does not appear to decrease with longer durations of each refreshing step. A Bayesian ANOVA on mean recall score with Number of refreshing steps (1, 2, or 3) and Duration of refreshing steps (333, 500, or 1,000 ms) revealed that the best model of the data was the null model (BF_01_ of 4.68 *against* the main effect of Number of refreshing steps, BF_01_ of 10.29 *against* the main effect of Duration of refreshing steps, and BF_01_ of 5633 against the full model including both main effects as well as their interaction; η^2^ < 0.01 associated with the main effect of Number of refreshing steps, with the main effect of Duration of refreshing steps, and with the interaction effect). Considering recall error for refreshed items only when they were last cued in the first or the second half of the cue sequences gave similar results (see Supplementary Analyses 1 and 2 (OSM)).

Finally, a set of one-sided, paired t-tests was run, to test the frequency and duration hypotheses more directly. Similar to what we did in Experiments 1 and 2, the frequency hypothesis was tested in three t-tests, examining whether recall error was smaller for items that had been refreshed more often while keeping the total duration of refreshing constant. This showed inconclusive evidence when comparing items that were refreshed twice for 500 ms versus once for 1,000 ms (BF_01_ = 1.49 for the Null; d = 0.18) and moderate evidence against the effect when comparing items that were refreshed three times for 333 ms versus twice for 500 ms (BF_01_ = 3.87 for the Null; d = 0.09). When comparing the most extreme difference, the t-test showed moderate evidence that refreshing items three times for 333 ms resulted in smaller recall error than refreshing items once for 1,000 ms (BF_10_ = 3.42; d = 0.28). When only considering recall error for items that were last cued by the fourth, fifth, or sixth cue, the three t-tests mainly showed evidence against the frequency hypothesis (BF_01_ = 4.27, BF_01_ = 4.64, and BF_01_ = 2.68, for the three aforementioned tests, respectively; d = 0.08, d = 0.07, and d = 0.12, respectively). The duration hypothesis was tested in nine t-tests, testing whether recall error decreased when the duration of refreshing steps was increased (1) from 333 to 500 ms, (2) from 500 to 1,000 ms, and (3) from 333 to 1,000 ms, for 1-refreshing, 2-refeshing, and 3-refreshing items, respectively. As can be seen in Table 1, all tests revealed evidence against the predicted effects under the duration hypothesis. Thus, even when the duration of refreshing steps was increased from 333 to 500 ms (i.e., within the 500-ms window), our data did not support the notion that spending more time in the focus of attention results in larger memory boosts.

**Table 1:**
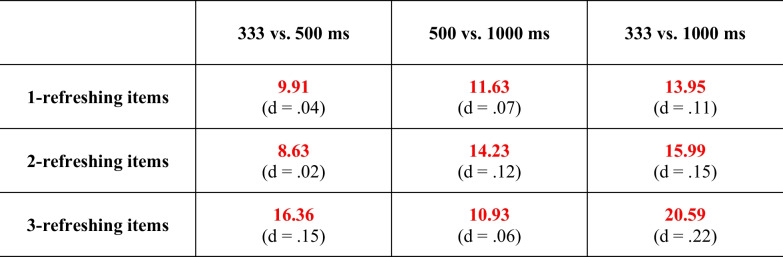
Evidence in the data against (in red) a memory boost (i.e., decrease in recall error) when increasing the duration of refreshing steps. Bayes factors are from paired, one-sided t-tests. The corresponding effect sizes (d) are shown in parentheses

### Discussion

In Experiment 3, there was some evidence for the notion that recall performance is affected by the number of times an item has been refreshed. As such, the results of Experiment 3 were more in line with the results of Experiment 1 than with the results of Experiment 2, suggesting that it was indeed the inclusion of very short refreshing steps of 250 ms that discouraged the use of refreshing cues in Experiment 2. However, when we aimed to assess the contribution of the number of refreshing steps versus the duration of each refreshing step, we did not find evidence for either. As in Experiment 1, varying the duration of the refreshing steps was not found to affect recall performance. Unlike Experiment 1, however, we no longer found evidence for a main effect of refreshing frequency when the condition of 0-refreshing was not included in the analysis. It thus seems that the overall effect of refreshing frequency in Experiment 3 was largely driven by the difference in recall performance between items that had been refreshed at least once versus items that had not been refreshed (see also Supplementary Analysis 3 (OSM)).

A set of t-tests confirmed that there is strong evidence in the data against the predictions of the duration hypothesis, together with some evidence for the predictions of the selection hypothesis. In particular, contrary to the duration hypothesis, refreshing an item for 1,000 ms did not improve its recall performance, relative to refreshing it for 333 or 500 ms (and these last two were not different either). As for the selection hypothesis, whereas recall performance was not found to be better when comparing either items that were refreshed three times for 333 ms versus twice for 500 ms, or items that were refreshed twice for 500 ms versus once for 1,000 ms, there was some evidence for better recall when comparing the two extremes. That is, refreshing an item three times for 333 ms did result in better recall than refreshing an item once for 1,000 ms. Thus, even though the evidence for the effect of refreshing frequency is much weaker than what we observed in Experiment 1, the findings of Experiment 3 seem to corroborate the overall conclusions of Experiment 1, with some evidence for the selection hypothesis but not for the duration hypothesis.

## General discussion

Our aim for the current study was to advance the understanding of how refreshing results in better working memory for the refreshed information. We proposed that refreshing could boost memory in either a constant (time-independent) or gradual (time-dependent) fashion. Specifically, we reasoned that, if the beneficial effect of refreshing stems from the act of being selected for attentional focusing, then the main determinant of memory performance is the number of times an item has been refreshed. In contrast, the beneficial effect of refreshing could stem from spending time in the focus of attention, resulting in a gradual, time-dependent memory boost. Thus, according to the duration hypothesis, the memory boost induced by refreshing would depend on the amount of time an item has been the object of refreshing, rather than the number of times the item has been selected for refreshing. Our results indicate that, when an effect of cued refreshing is observed on memory performance, the memory boost appears to be constant rather than time-dependent. In what follows, we discuss the implications of these findings for our understanding of refreshing.

### How does instructed refreshing boost memory?

Instructing and guiding the use of refreshing through the presentation of refreshing cues is one direct way to examine the consequences and operation of refreshing. Doing so, we have four observations that can help us to better understand the memory boost that results from cued refreshing. First, we found that memory performance generally increased as items were cued to be refreshed more often, thereby replicating the findings of Souza and colleagues as well as extending them to refreshing steps that have durations other than 500 ms.

Second, the effect of the number of refreshing steps is constrained to certain task parameters. In Experiment 1, we observed clear evidence for a refreshing frequency effect, both when the refreshing steps had a duration of 500 ms (as usually implemented in the literature) and when we doubled the duration to 1,000 ms. This mirrors the recent finding of Loaiza and Souza (2022), who also included refreshing steps of 500 and 1,000 ms (albeit not controlling for total duration as done here). Hence, increasing the duration of the cues beyond 500 ms does not help, but also does not change the main findings. In our Experiment 2, however, the effect of cued refreshing disappeared entirely when we included very short cue durations (250 ms). This suggests that reducing the refreshing duration by half may have discouraged participants to follow and use the refreshing cues altogether. Accordingly, in Experiment 3, we used somewhat longer refreshing steps, intermixing cue durations of 333 ms, 500 ms, and 1,000 ms, and we again found evidence for an effect of cued refreshing on memory performance. This indicates that, in Experiment 3, participants were following and using the refreshing cues. However, unlike Experiment 1, the effect of cued refreshing in Experiment 3 was mainly driven by the difference between items that had never been refreshed and items that had been refreshed, without a credible effect of the number of refreshing steps (1 to 3). It is possible that participants only used some of the refreshing cues in Experiment 3, either because there were many more cues to follow (six cues in Experiment 3, instead of four cues in Experiment 1) or because the retention interval was longer (4 s in Experiment 3, instead of 3 s in Experiment 1). Hence, taken together, our experiments do provide evidence for a beneficial effect of refreshing on memory performance, but they also point to limitations on how to implement the cued refreshing procedure. In particular, our results suggest that the use of 500-ms cues is the most efficient way to manipulate refreshing, and that very long cue sequences can discourage people from following all cues, blurring the distinction between items cued to be refreshed 1, 2, and 3 times.

Third, we found no evidence for the predictions of the duration hypothesis. Lengthening the duration of the refreshing steps did not result in better memory performance in any of our experiments. This fits well with the recent results of Loaiza and Souza (2022), who did not find a beneficial effect of longer refreshing steps either. In that study, there was even some evidence for worse memory performance when longer refreshing steps were used.^1^ It is worth noting that in the present studies, cues with different durations were intermixed within the same trial in order to keep the retention interval always constant across conditions. Yet, this design choice implies that participants could not anticipate the cue duration and prepare for making the best use of the time available. In Loaiza and Souza (2022), in contrast, cue duration was varied between blocks of trials, so that participants could fully prepare for the duration of the cue and try to make the best use of the time available, with the downside that this led to different total retention intervals across blocks (to compensate for this, the authors included control conditions with variations in the total retention interval). Yet, both sets of experiments converge with regard to the lack of a benefit for increasing cue duration. Therefore, unpredictability and lack of preparation are unlikely to account for our evidence against the duration hypothesis.

One could argue that we manipulated the time available for refreshing overall rather than the duration of each refreshing step.^2^ We instructed participants to refresh the cued items for the amount of time the cue was onscreen, but we cannot ensure that participants followed our instructions closely in all trials. It is possible that participants sneaked in some refreshing of uncued items, especially during refreshing steps longer than 500 ms. If that is the case, it may explain why we did not find better memory performance for longer refreshing durations. The use of refreshing cues allows for a more direct manipulation of refreshing than manipulations of cognitive load, but additional controls may be needed in future experiments to ensure that participants are following the instructions closely and consistently. One possibility would be to use eye tracking to monitor cue use. Loaiza and Souza (2022) observed that looking back at uncued locations during the instructed refreshing procedure was associated with better recall of 0-refreshed items, in line with the possibility that participants were sneaking in uninstructed refreshings during the retention interval. Future experiments could also consider a more gradual, and perhaps random, manipulation of the duration of each refreshing step, rather than a few selected durations.^3^ Overall, while our data do not provide any evidence for the duration hypothesis, we cannot rule out the possibility that a different experimental set-up might yield some evidence in its favor.

Papers modeling the refreshing process have implemented refreshing as a boost that develops over time (Gauvrit & Mathy, 2018; Lemaire & Portrat, 2018; Oberauer & Lewandowsky, 2011), with studies varying on the specification of the function that describes this relation (e.g., a logarithmic function). A logarithmic function describes a quick ascending but decelerating process, which would be consistent with refreshing providing a rapid boost (for example, in the first 250 ms), followed by a quite shallow additional gain in activation over time that may be difficult to reliably measure.^4^ Our results cannot rule out this possibility.

Fourth, and finally, when a beneficial effect of cued refreshing was observed in our study, the memory boost appeared to be a constant phenomenon, as predicted by the selection hypothesis. Thus, our results indicate that each time an item is selected for refreshing, it receives a fixed memory boost, and thus, items that are refreshed more often can accumulate more of these boosts, resulting in better memory performance. Overall, the current pattern of results is thus more in line with the notion that the benefit of refreshing depends on the number of times an item has been selected for attentional focusing, rather than on the time during which an item has remained the object of attentional focusing. Our observation in working memory echoes the beneficial effect of retrieval practice in long-term memory (e.g., Roediger & Karpicke, 2006), with both effects suggesting that memory performance improves through repeated engagement (repeated testing, or repeated refreshing).

## Supplementary information

Below is the link to the electronic supplementary material.

Supplementary file1 (DOCX 59 KB)

## Data Availability

The data for all experiments are available on the Open Science Framework (OSF; https://osf.io/frbn8/). None of the materials are available and none of the experiments was preregistered.

## Footnotes

1 Note that these results were not known when the current experiments were designed and ran.

2 We thank an anonymous reviewer for this suggestion.

3 We thank an anonymous reviewer for this suggestion.

4 We thank an anonymous reviewer for this suggestion.

## Code availability

The code to run the analyses is not available.

## Declarations

### Author Note

Most of the work was done when Alessandra S. Souza was at the University of Zurich.
