# Supplementary material for: The effect of instructed refreshing on working memory: Is the memory boost a function of refreshing frequency or refreshing duration?
Source: Mem Cognit. 2024 Dec 2;53(5):1510–22. doi: 10.3758/s13421-024-01666-w (PMC12307513; doi:10.3758/s13421-024-01666-w)
Supplement: Supplementary file 1 — Supplementary file1 (DOCX 59 KB) [file 13421_2024_1666_MOESM1_ESM.docx]

**Supplementary materials**

**Table S1**

*The ten possible cue sequences used in Experiment 1, with their timings (cues in red were presented for 1000 ms, cues in blue were presented for 500 ms). The letters A-F represent the six memory items on any given trial. In some sequences, four different memory items were randomly selected to be refreshed (A, B, C, and D, in cue sequences 1 and 2); in other sequences, three different memory items were randomly selected to be refreshed (A, B, and C, in cue sequences 3 through 8), or two different memory items were randomly selected to be refreshed (A and B, in cue sequences 9 and 10).*

|  | **0-refreshing** | **1-refreshing** | | **2-refreshing** | |
| --- | --- | --- | --- | --- | --- |
| **Cue Sequence** |  | **1*500 ms** | **1*1000 ms** | **2*500 ms** | **2*1000 ms** |
| (1) A-B-C-D | E-F | B-D | A-C | -- | -- |
| (2) A-B-C-D | E-F | A-C | B-D | -- | -- |
| (3) A-B-C-B | D-E-F | -- | A-C | B | -- |
| (4) A-B-C-B | D-E-F | A-C | -- | -- | B |
| (5) A-B-A-C | D-E-F | -- | B-C | A | -- |
| (6) A-B-A-C | D-E-F | B-C | -- | -- | A |
| (7) A-B-C-A | D-E-F | -- | B-C | A | -- |
| (8) A-B-C-A | D-E-F | B-C | -- | -- | A |
| (9) A-B-A-B | C-D-E-F | -- | -- | B | A |
| (10) A-B-A-B | C-D-E-F | -- | -- | A | B |

**Table S2**

*The 36 possible cue sequences used in Experiment 3, with their timings (cues in red were presented for 1000 ms, cues in blue were presented for 500 ms, and cues in green were presented for 333 ms). The letters A-F represent the six memory items on any given trial. In all sequences, three different memory items were randomly selected to be refreshed (A, B, and C).*

|  |  | **0-refreshing** | **1-refreshing** | | | **2-refreshing** | | | **3-refreshing** | | |
| --- | --- | --- | --- | --- | --- | --- | --- | --- | --- | --- | --- |
| **Potential targets** | **Cue Sequence** |  | **1*333 ms** | **1*500 ms** | **1*1000 ms** | **2*333 ms** | **2*500 ms** | **2*1000 ms** | **3*333 ms** | **3*500 ms** | **3*1000 ms** |
|  | A-B-A-B-C-B | D-E-F | -- | C | -- | -- | -- | A | -- | B | -- |
|  | A-B-C-B-A-B | D-E-F | -- | C | -- | -- | -- | A | -- | B | -- |
| *0 refreshing* | A-B-C-B-C-B | D-E-F | -- | A | -- | -- | -- | C | -- | B | -- |
| *1*500 ms* | A-B-A-C-B-A | D-E-F | -- | C | -- | -- | -- | B | -- | A | -- |
| *2*1000 ms* | A-B-A-B-C-A | D-E-F | -- | C | -- | -- | -- | B | -- | A | -- |
| *3*500 ms* | A-B-C-A-B-A | D-E-F | -- | C | -- | -- | -- | B | -- | A | -- |
|  | A-B-C-A-C-A | D-E-F | -- | B | -- | -- | -- | C | -- | A | -- |
|  | A-B-A-B-A-C | D-E-F | -- | C | -- | -- | -- | B | -- | A | -- |
|  | A-B-A-C-A-C | D-E-F | -- | B | -- | -- | -- | C | -- | A | -- |
|  | A-B-A-B-C-B | D-E-F | -- | -- | C | -- | -- | A | B | -- | -- |
|  | A-B-C-B-A-B | D-E-F | -- | -- | C | -- | -- | A | B | -- | -- |
|  | A-B-C-B-C-B | D-E-F | -- | -- | A | -- | -- | C | B | -- | -- |
| *0 refreshing* | A-B-A-C-B-A | D-E-F | -- | -- | C | -- | -- | B | A | -- | -- |
| *1*1000 ms* | A-B-A-B-C-A | D-E-F | -- | -- | C | -- | -- | B | A | -- | -- |
| *2*1000 ms* | A-B-C-A-B-A | D-E-F | -- | -- | C | -- | -- | B | A | -- | -- |
| *3*333 ms* | A-B-C-A-C-A | D-E-F | -- | -- | B | -- | -- | C | A | -- | -- |
|  | A-B-A-B-A-C | D-E-F | -- | -- | C | -- | -- | B | A | -- | -- |
|  | A-B-A-C-A-C | D-E-F | -- | -- | B | -- | -- | C | A | -- | -- |
|  | A-B-A-B-A-C | D-E-F | C | -- | -- | B | -- | -- | -- | -- | A |
| *0 refreshing* | A-B-A-C-A-C | D-E-F | B | -- | -- | C | -- | -- | -- | -- | A |
| *1*333 ms* | A-B-A-C-A-B | D-E-F | C | -- | -- | B | -- | -- | -- | -- | A |
| *2*333 ms* | A-B-A-B-C-B | D-E-F | C | -- | -- | A | -- | -- | -- | -- | B |
| *3*1000 ms* | A-B-C-B-C-B | D-E-F | A | -- | -- | C | -- | -- | -- | -- | B |
|  | A-B-C-B-A-B | D-E-F | C | -- | -- | A | -- | -- | -- | -- | B |
|  | A-B-A-C-B-C | D-E-F | -- | -- | -- | -- | B-C | A | -- | -- | -- |
|  | A-B-C-A-B-C | D-E-F | -- | -- | -- | -- | B-C | A | -- | -- | -- |
|  | A-B-C-B-A-C | D-E-F | -- | -- | -- | -- | B-C | A | -- | -- | -- |
|  | A-B-C-B-C-A | D-E-F | -- | -- | -- | -- | B-C | A | -- | -- | -- |
| *0 refreshing* | A-B-A-C-B-C | D-E-F | -- | -- | -- | -- | A-C | B | -- | -- | -- |
| *2*500 ms* | A-B-C-A-B-C | D-E-F | -- | -- | -- | -- | A-C | B | -- | -- | -- |
| *2*1000 ms* | A-B-C-B- A-C | D-E-F | -- | -- | -- | -- | A-C | B | -- | -- | -- |
|  | A-B-C-B-C-A | D-E-F | -- | -- | -- | -- | A-C | B | -- | -- | -- |
|  | A-B-A-C-B-C | D-E-F | -- | -- | -- | -- | A-B | C | -- | -- | -- |
|  | A-B-C-A-B-C | D-E-F | -- | -- | -- | -- | A-B | C | -- | -- | -- |
|  | A-B-C-B-A-C | D-E-F | -- | -- | -- | -- | A-B | C | -- | -- | -- |
|  | A-B-C-B-C-A | D-E-F | -- | -- | -- | -- | A-B | C | -- | -- | -- |

**Supplementary Analysis 1**

When there are four refreshing cues, a 2-refreshing item can be last refreshed in cue position 3 or 4, whereas a 1-refreshing item can be last refreshed in cue position 1, 2, 3, or 4. Hence, 2-refreshing items not only benefit from two refreshing steps, but also have a shorter delay between cuing and testing overall. To minimize the impact of cue position and the delay between cuing and testing, we reran the analyses reported in the main text for Experiments 1 and 2, this time including only refreshed items last cued by the third or fourth cue. For Experiment 3, in which there were 6 refreshing cues, we reran the analyses reported in the main text including only refreshed items last cued by the fourth, fifth, or sixth cue.

**Experiment 1.** First, we ran BANOVA on mean recall error with Refreshing frequency (0, 1, or 2 refreshing steps) as within-subject predictor. This revealed strong evidence for a main effect of the number of refreshing steps on recall (BF_10_ = 220.05; η^2^ = .27). Next, we ran a BANOVA on mean recall error with Number of refreshing steps (1 or 2) and Duration of refreshing steps (500 vs. 1000 ms). The best model was the Number of refreshing steps-only model (BF_10_ = 12.22; η^2^ = .10). There was modest evidence *against* including the variable Duration of refreshing steps (BF_01_ = 4.85; η^2^ < .01), and the best model was preferred over the full model including both main effects as well as their interaction by a factor of 18.47 (η^2^ < .01 associated with the interaction effect).

**Experiment 2.** A BANOVA on mean recall error with Refreshing frequency (0, 1, or 2 refreshing steps) as within-subject predictor revealed that the best model of the data was the null model, with some weak evidence against the main effect of Refreshing frequency (BF_01_ = 1.56; η^2^ = .08). Next, a BANOVA on mean recall error with Number of refreshing steps (1 or 2) and Duration of refreshing steps (250 vs. 500 ms) revealed that the best model of the data was the null model (BF_01_ of 5.15 against the main effect of Number of refreshing steps, and BF_01_ of 3.44 against the main effect of Duration of refreshing steps, and BF_01_ of 46.39 against the full model including both main effects as well as their interaction; η^2^ <.01 associated with the main effect of Number of refreshing steps, and η^2^ = .01 associated with the main effect of Duration of refreshing steps and with the interaction effect).

**Experiment 3.** A BANOVA with Refreshing frequency (0, 1, 2, or 3 refreshing steps) as within-subject predictor showed strong evidence for a main effect of the number of refreshing steps on recall error (BF_10_ = 201.39; η^2^ = .10). A BANOVA on mean recall score with Number of refreshing steps (1, 2, or 3) and Duration of refreshing steps (333, 500, or 1000 ms) revealed that the best model of the data was the null model (BF_01_ of 12.92 against the main effect of Number of refreshing steps, BF_01_ of 35.20 against the main effect of Duration of refreshing steps, and BF_01_ of 13134 against the full model including both main effects as well as their interaction; η^2^ <.01 associated with the main effect of Number of refreshing steps, with the main effect of Duration of refreshing steps, and with the interaction effect).

**Supplementary Analysis 2**

We ran additional analyses to explore whether the effects of refreshing frequency and refreshing duration are found more strongly when only including refreshed items last cued in the first half of the cue sequence (as suggested by an anonymous reviewer). In Experiments 1 and 2, we could not examine the effect of refreshing frequency this way, because items that are refreshed more than once are always last cued in the second half of the cue sequence. However, the effect of refreshing duration could be examined. In particular, in Experiments 1 and 2, we examined recall error for items that were refreshed once and for which the refreshing cue was presented in cue position 1 or 2. In Experiment 1, a one-sided paired t-test showed evidence against smaller recall error for items refreshed once for 1000 ms compared to items refreshed once for 500 ms (BF_01_ = 4.22 for the Null; d = .04); in Experiment 2, a one-sided paired t-test showed evidence against smaller recall error for items refreshed once for 500 ms compared to items refreshed once for 250 ms (BF_01_ = 6.11 for the Null; d = .04).

In Experiment 3, we could examine the effects of both refreshing frequency and refreshing duration in this way. This meant only including items that were refreshed once or twice (because items that are refreshed more than twice were always last cued in the second half of the cue sequence). Fourteen participants had at least one empty cell, and their data was entirely removed from the dataset for this specific analysis. A BANOVA on mean recall score with Number of refreshing steps (1 or 2) and Duration of refreshing steps (333, 500, or 1000 ms) revealed that the best model of the data was the null model (BF_01_ of 7.55 against the main effect of Number of refreshing steps, BF_01_ of 6.89 against the main effect of Duration of refreshing steps, and BF_01_ of 417.93 against the full model including both main effects as well as their interaction; η^2^ <.01 associated with the main effect of Number of refreshing steps, η^2^ = .01 associated with the main effect of Duration of refreshing steps, and η^2^ <.01 associated with the interaction effect).

**Supplementary Analysis 3**

Additional t-tests were conducted to further analyze the main effect of refreshing frequency observed in Experiments 1 and 3. In Experiment 1, paired one-sided t-tests were used to test whether recall error was smaller for items refreshed once (regardless of the duration of the refreshing step) compared to items that were not refreshed, and whether recall error was smaller for items refreshed twice compared to items refreshed once (regardless of the duration of the refreshing steps). There was modest evidence for the decrease in recall error from 0 to 1 refreshing step (BF_10_ = 4.53; d = .44) and strong evidence for the decrease in recall error from 1 to 2 refreshing steps (BF_10_ = 22.65; d = .58). In Experiment 3, paired one-sided t-tests were used to test whether recall error was smaller for items refreshed once (regardless of the duration of the refreshing step) compared to items that were not refreshed, whether recall error was smaller for items refreshed twice compared to items refreshed once (regardless of the duration of the refreshing steps), and whether recall error was smaller for items refreshed three times compared to items refreshed twice (regardless of the duration of the refreshing steps). There was clear evidence for the decrease in recall error from 0 to 1 refreshing step (BF_10_ = 9.62; d = .34), very weak evidence against the decrease in recall error from 1 to 2 refreshing steps (BF_01_ = 1.47; d = .18), and rather strong evidence against the decrease in recall error from 2 to 3 refreshing steps (BF_01_ = 7.02; d = .01).

Additional t-tests were also run to further test the duration hypothesis in Experiments 1 and 2, by testing for the predicted positive effect of refreshing duration, while keeping the number of refreshing steps constant. Specifically, in Experiment 1, the duration hypothesis was tested in two ways: (1) by testing whether recall error decreased when the duration of the single refreshing step of a 1-refreshing item was increased from 500 to 1000 ms, and (2) by testing whether recall error decreased when the duration of the two refreshing steps of a 2-refreshing item was increased from 500 to 1000 ms. There was modest evidence against both effects (BF_01_ = 5.83 and 6.25 in favor of the Null, respectively; d = .03 and d = .05, respectively). In Experiment 2, recall error was not smaller when the duration of the single refreshing step of a 1-refreshing item was increased from 250 to 500 ms (BF_01_ = 6.48 for the Null; d = .06), and the data remained inconclusive as to whether recall error decreased when the duration of the two refreshing steps of a 2-refreshing item was increased from 250 to 500 ms (BF_10_ = 1.34; d = .31). This contrasts with the prediction of the duration hypothesis.
